# Sodium Citrate Increases Expression and Flux of Mg^2+^ Transport Carriers Mediated by Activation of MEK/ERK/c-Fos Pathway in Renal Tubular Epithelial Cells

**DOI:** 10.3390/nu10101345

**Published:** 2018-09-20

**Authors:** Yui Takashina, Aya Manabe, Hajime Hasegawa, Toshiyuki Matsunaga, Satoshi Endo, Akira Ikari

**Affiliations:** 1 Laboratory of Biochemistry, Department of Biopharmaceutical Sciences, Gifu Pharmaceutical University, Gifu 501-1196, Japan; 145037@gifu-pu.ac.jp (Y.T.); 135072@gifu-pu.ac.jp (A.M.); matsunagat@gifu-pu.ac.jp (T.M.); sendo@gifu-pu.ac.jp (S.E.); 2 Saitama Medical Center, Saitama Medical University, Saitama 350-8550, Japan; hase2126@saitama-med.ac.jp

**Keywords:** alkalization, hypomagnesemia, NADPH, TRPM6

## Abstract

A chronic magnesium deficiency may be one of the causes of lifestyle-related diseases such as hypertension and diabetes. Serum Mg^2+^ concentration is strictly controlled by the reabsorption pathway in the renal tubules, but little is known about how Mg^2+^ reabsorption is upregulated. We searched for food compounds which can increase the expression levels of Mg^2+^ transport carriers including transient receptor potential melastatin 6 (TRPM6) channel and cyclin M2 (CNNM2). Sodium citrate (SC) increased the mRNA levels of TRPM6 and CNNM2 in renal tubular epithelial NRK-52E cells. The SC-induced elevation of TRPM6 was inhibited by U0126, a mitogen-activated protein kinase kinase (MEK) inhibitor, but the CNNM2 was not. SC increased the levels of p-ERK1/2 and p-c-Fos, which were inhibited by U0126. SC induced alkalization of culture medium. Both SC and alkalization enhanced Mg^2+^ influx, which was inhibited by U0126 and introduction of TRPM6 siRNA. The reporter activity of TRPM6 was increased by SC and alkalization, which was suppressed by mutation in an AP-1-binding site. The SC-induced elevation of p-ERK1/2 and p-EGFR was inhibited by diphenylene iodonium, a nicotinamide adenine dinucleotide phosphate (NADPH) oxidase inhibitor, and erlotinib, an epidermal growth factor receptor (EGFR) tyrosine kinase inhibitor. SC did not change the level of acetyl histone H3, but increased the association of c-Fos with the promoter region of TRPM6. These results suggest that SC increases TRPM6 expression and Mg^2+^ influx mediated by the activation of NADPH oxidase and an EGFR/ERK/c-Fos pathway in the renal tubules.

## 1. Introduction

Magnesium ion (Mg^2+^) is the fourth most abundant metal in the body and is involved in a variety of physiological functions. Over 300 enzymes including oxidative phosphorylation and glycolysis require Mg^2+^ for their activity. Mg^2+^ deficiency may be a risk factor for lifestyle-related diseases such as hypertension, diabetes mellitus, and hyperlipidemia [1,2,3]. In addition, Mg^2+^ is necessary to protect individuals from physical and mental stresses [4,5]. The individuals under excessive stress conditions may have broken balance between Mg^2+^ intake and waste, leading to the state of chronic Mg^2+^ deficiency. However, Mg^2+^ deficiency is difficult to be actualized because stored Mg^2+^ is released from bone and muscle to blood under mild Mg^2+^ depletion.

The recommended daily intake of Mg^2+^ for a normal adult is about 300–400 mg and 30–50% is absorbed in the intestine (Net intake is about 100 mg/day). The intestinal absorption of Mg^2+^ is poorly regulated by Mg^2+^-dependent carriers and depends on the amount of Mg^2+^ intake [6]. The absorption rate is enhanced up to 80% of dietary Mg^2+^ when Mg^2+^ intake is low [7]. Serum Mg^2+^ is filtrated by renal glomeruli and then reabsorbed along the nephron. The reabsorption pathway and rate are different in each segment of nephron. The proximal tubule and the thick ascending limb of Henle’s loop (TAL) are sites of passive paracellular Mg^2+^ reabsorption, whereas the distal convoluted tubule (DCT) is the main site of active transcellular Mg^2+^ reabsorption [8]. The majority (over 60%) of total filtrated Mg^2+^ is reabsorbed in the TAL. The reabsorption rate in the DCT cells is lower than 5%, but it is critically involved in the fine-tuning of serum Mg^2+^ concentration. The renal reabsorption process may be a target for preventing Mg^2+^ deficiency.

In the DCT, the negative plasma membrane potential provides transcellular activity of Mg^2+^ transport from the lumen to cells. On the other hand, Na^+^ gradient caused by the Na^+^/K^+^-ATPase may be used to transport Mg^2+^ from the cells to blood. Transient receptor potential melastatin 6 (TRPM6) Mg^2+^ channel is expressed at the apical membrane of DCT. The *TRPM6* gene has been identified as the causative gene for a rare autosomal recessive disorder, hypomagnesemia with secondary hypocalcemia [9,10]. The tissue distribution of TRPM6 is restricted to the apical membrane of DCT and intestine [11]. In contrast, TRPM7, a closest homologue of TRPM6, is expressed ubiquitously in a broad spectrum of tissues. Cyclin M2 (CNNM2, previously known as ACDP2) is exclusively expressed at the basolateral membrane of DCT and is supposed to function as an Mg^2+^ transporter or modulator of other Mg^2+^ carriers [12]. Mutations in the *CNNM2* gene are identified in patients with unexplained dominant hypomagnesemia [13]. Both TRPM6 and CNNM2 expression is responsive to Mg^2+^ intake in mice [14,15]. These Mg^2+^ carriers may play an important role in transcellular Mg^2+^ reabsorption.

A randomized, double-blind study showed that supplementation of Mg-citrate induces greater mean serum Mg^2+^ than Mg-oxide and Mg-amino acid chelate [16]. The difference of Mg^2+^ bioavailability is suggested to be due to their solubility to water and intestinal absorption [17], but the detail mechanisms remain unknown. The relationship between hypomagnesemia and hypocitraturia has been reported in a patient with familial hypomagnesemia and hypercalciuria with nephrocalcinosis, who have decreased serum Mg^2+^, increased urinary excretion of Mg^2+^, and low citrate excretion [18]. While citrate may be involved in the regulation of renal Mg^2+^reabsorption, there is no report about whether citrate increases the expression levels of Mg^2+^ carriers.

In the present study, we found that sodium citrate (SC) increases the mRNA and protein levels of TRPM6 in renal tubular epithelial NRK-52E cells. The upregulatory mechanism of TRPM6 was examined using specific inhibitors of intracellular signaling pathways. In addition, the effect of SC on intracellular pH, Mg^2+^ influx, TRPM6 promoter activity, and binding of transcription factor to the promoter region of TRPM6 to clarify the molecular mechanism. SC may be useful to exaggerate the Mg^2+^ reabsorption in the kidney and prevent chronic Mg^2+^ deficiency.

## 2. Experimental Section

### 2.1. Materials

Calcium citrate (CC) and SC were purchased from Wako Pure Chemical Industries (Osaka, Japan). Diphenylene iodonium (DPI), Erlotinib, 5-(*N*-ethyl-*N*-isopropyl)amiloride (EIPA), 5-oxazolecarboxylic acid, 2-[5-[2-[(acetyloxy)methoxy]-2-oxoethoxy]-6-[bis[2-[(acetyloxy)methoxy]-2-oxoethyl]amino]-2-benzofuranyl]-(acetyloxy)methyl ester (Mag-fura-2 AM), and U0126 were from Cayman Chemical (Ann Arbor, MI, USA), Adooq BioScience (Irvine, CA, USA), Enzo Life Sciences (Farmingdale, NY, USA), ThermoFisher Scientific (Waltham, MA, USA), LC Laboratories (Woburn, MA, USA), respectively. Anti-actin, anti-phosphorylated ERK1/2 (p-ERK1/2), anti-c-Fos, and anti-p22phox antibodies were from Santa Cruz Biotechnology (Santa Cruz, CA, USA). Anti-p-EGFR, anti-ERK1/2, and anti-c-Fos antibodies were from Cell Signaling Technology (Beverly, MA, USA). Anti-EGFR, anti-acetyl-histone H3, and histone H3 antibodies were from GeneTex (Hsinchu, Taiwan). Anti-CNNM2, anti-NADPH oxidase (NOX) 4, and anti-TRPM7 antibodies were from Bioss Antibodies (Woburn, MA, USA), ProteinTech (Rosemont, IL, USA), and Novus Biologicals (Littleton, CO, USA), respectively. Peroxidase-labeled antibodies to mouse IgG, rabbit IgG, and goat IgG were from SeraCare Life Sciences (Milford, MA, USA). All other reagents were of the highest grade of purity available.

### 2.2. Cell Culture

NRK-52E cells (IFO50480), derived from normal rat renal tubules, were obtained from Japanese Collection of Research Biosciences (Osaka, Japan). The cells were grown in Dulbecco’s modified Eagle’s medium as described previously [19]. Cells were cultured for 24 h in the medium without fetal bovine serum before experiments. When the pH of culture medium was adjusted to alkali (pH 7.6 and 7.8), NaOH was added into the Hepes-buffered medium (25 mM Hepes). Negative or TRPM6 siRNA was transfected in the cells as described previously [20]. Control cells were treated with dimethyl sulfoxide (DMSO) as a vehicle.

### 2.3. RNA Isolation and Quantitative Real-Time Reverse-Transcription Polymerase Chain Reaction (PCR)

Total RNA was isolated from cells using TRI reagent (Sigma-Aldrich, St. Louis, MO, USA). Reverse transcription was carried out using a ReverTra Ace qPCR RT kit (Toyobo Life Science, Osaka, Japan). Quantitative real-time PCR was performed using a KOD SYBR qPCR Mix (Toyobo Life Science, Osaka, Japan). The reaction conditions were an initial 60 s denaturation at 95 °C, followed by 40 cycles of amplification (15 s of denaturation at 95 °C and 60 s of extension at 60 °C). Primers used for PCR are shown in Table 1. The threshold cycle (Ct) for each PCR product was calculated by the instrument’s software, and Ct values obtained for TRPM6, CNNM2, and TRPM7 were normalized by dividing the Ct values obtained for β-actin. The resulting ΔCt values were then used to calculate the relative change in the mRNA expression as a ratio (*R*) according to the equation, *R* = 2^−(Ct(drug treatment) −ΔCt(vehicle))^.

### 2.4. Preparation of Cytoplasmic Extracts and Western Blotting

Cytoplasmic extracts, which include plasma membrane and cytosolic proteins, were prepared as described previously [19]. The aliquots were applied to sodium dodecyl sulfate-polyacrylamide gel electrophoresis (SDS-PAGE) and blotted onto a polyvinylidene fluoride (PVDF) membrane. After blocking with 4% Block Ace (Dainippon Sumitomo Pharma, Osaka, Japan) at room temperature for 0.5 h, the membrane was incubated with each primary antibody (1:1000 dilution) at 4 °C for 16 h, followed by a peroxidase-conjugated secondary antibody (1:3000 dilution) at room temperature for 1.5 h. The PVDF membrane was blocked with 2% bovine serum albumin when we used phospho-specific antibodies. Finally, the membrane were incubated in EzWestLumi plus (ATTO Corporation, Tokyo, Japan) and scanned using a C-DiGit Blot Scanner (LI-COR Biotechnology, Lincoln, NE, USA). Band density was quantified using ImageJ software (National Institute of Health software (Bethesda, MD, USA). β-actin was used for normalization of cytoplasmic proteins.

### 2.5. Immunoprecipitation

In immunoprecipitation assay, the cytoplasmic extracts were incubated with protein G sepharose beads and anti-NOX4 antibody at 4 °C for 16 h with gentle rocking. After centrifugation at 6000× *g* for 1 min, the pellet was washed four times with RIPA buffer. The immunoprecipitants were solubilized in a sample buffer for SDS-PAGE.

### 2.6. Luciferase Reporter Assay

The promoter region of the human *TRPM6* isoform a gene [NM_017662.4] was prepared as described previously [19]. A Renilla construct, pRL-TK vector (Promega, Madison, WI, USA), was used for normalizing transfection efficiency. Cells were transfected with plasmid DNA using HilyMax (Dojindo Laboratories, Kumamoto, Japan). After 48 h of transfection, luciferase activity was assessed using the Dual-Glo Luciferase Assay System (Promega, Madison, WI, USA). U0126 was added for the final 24 h before the luciferase assay. The luminescence of the firefly and renilla luciferase was measured using an AB-2270 Luminescencer Octa (Atto Corporation, Tokyo, Japan). The mutant of AP-1 binding site was previously generated [19].

### 2.7. Mg^2+^ Influx Assay

The change in [Mg^2+^]_i_ was determined using a Mg^2+^-sensitive fluorescent dye, Mag-fura 2 AM, as described previously [21,22]. MgCl_2_ (final concentration 1 mM) was added to the nominally Mg^2+^-free Hanks Balanced Salt Solution (HBSS) containing 137 mM NaCl, 5.4 mM KCl, 4.2 mM NaHCO_3_, 3 mM Na_2_HPO_4_, 0.4 mM KH_2_PO_4_, 5 mM Hepes, 1 mM CaCl_2_, and 10 mM glucose, immediately after the measurement start. [Mg^2+^]_i_ is represented as arbitrary units relative to a reference value measured at 0 s. The increase in fluorescence values of Mag-fura 2 for 150 s (ΔA.U.) was compared in each group.

### 2.8. Reactive Oxygen Species (ROS) Production

The production of ROS was determined using 2′,7′-dichlorofluorescin diacetate (DCFDA), a marker of a wide spectrum of ROS. Cells were incubated with 10 μM DCFDA at 37 °C for 30 min. After washing with HBSS twice, the fluorescence intensity of DCFDA was measured by Infinite F200 fluorescence microplate reader (Teca, Salzburg, Austria).

### 2.9. Chromatin Immunoprecipitation Assay

Cells were treated with 1% formaldehyde to crosslink the protein to DNA. Then, chromatin immunoprecipitation (ChIP) assays were performed using an EpiQuik Chromatin Immunoprecipitation kit (Epigentek, Farmingdale, NY, USA) as recommended by the manufacturer’s instructions. To co-immunoprecipitate the DNA, anti-c-Fos antibody was used. The eluted DNA was amplified by quantitative real-time PCR using the primer pairs -677S/-918A (Table 1). To confirm usage of the same amounts of chromatin in immunoprecipitation between groups, input chromatin was also used. ChIP data are represented as a % of input.

### 2.10. Statistics

Results are presented as mean ± standard error of the mean. Differences between groups were analyzed using a one-way analysis of variance, and corrections for multiple comparison were made using Tukey’s multiple comparison tests. Comparisons between two groups were made using student’s *t* test. Significant differences were assumed at *p* < 0.05.

## 3. Results

### 3.1. Increase in TRPM6 Expression by Sc and Calcium Citrate (CC)

**Figure 1:**
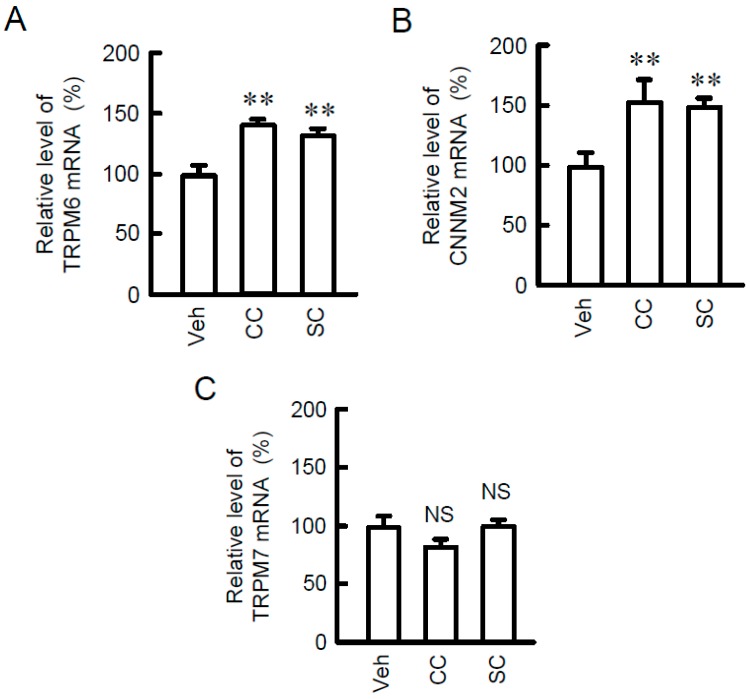
Increase in TRPM6 and CNNM2 expression by sodium citrate (SC) and calcium citrate (CC). NRK-52E cells were incubated with vehicle (dimethyl sulfoxide (DMSO), veh), 10 μM SC, or 10 μM CC for 24 h. After isolation of total RNA, quantitative real-time PCR was performed using primer pairs of TRPM6, CNNM2, TRPM7, and β-actin. The contents of TRPM6 (**A**), CNNM2 (**B**), and TRPM7 mRNAs (**C**) were represented as fold-increase over vehicle. *n* = 4. ** *p* < 0.01 and NS *p* > 0.05 compared with vehicle.

**Figure 2:**
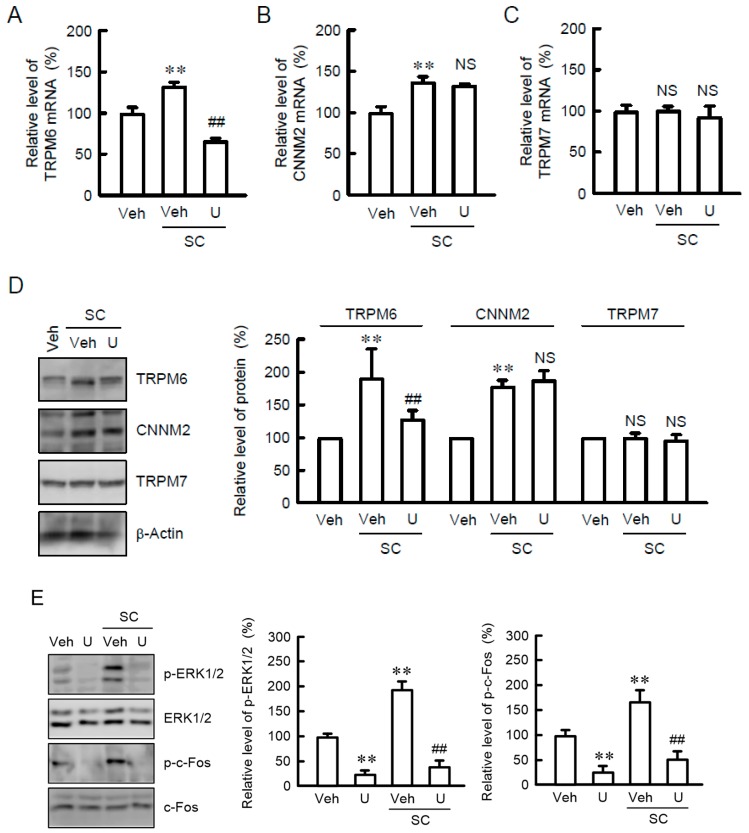
Inhibition of SC-induced TRPM6 expression by U0126. (**A**–**C**) NRK-52E cells were incubated with vehicle (veh), 10 μM SC, or 10 μM U0126 (U) for 24 h. Quantitative real-time PCR was performed using primer pairs of TRPM6, CNNM2, TRPM7, and β-actin. The contents of TRPM6, CNNM2, and TRPM7 mRNAs were represented as fold-increase over vehicle. (**D**) Cells were incubated with vehicle (veh), 10 μM SC, or 10 μM U0126 (U) for 24 h. Cytoplasmic extracts were immunoblotted with anti-TRPM6, anti-CNNM2, anti-TRPM7, or anti-β-actin antibody. The expression levels were represented relative to the values in vehicle. (**E**) Cells were incubated with vehicle (veh), 10 μM SC, or 10 μM U0126 (U) for 15 min. Cytoplasmic extracts were immunoblotted with anti-p-ERK1/2, anti-ERK1/2, anti-p-c-Fos, or anti-c-Fos antibody. The expression levels were represented relative to the values in veh. *n* = 4. ** *p* < 0.01 and NS *p* > 0.05 compared with vehicle. ^##^ *p* < 0.01 compared without U0126.

We recently found that NRK-52E cells constitutively express TRPM6, TRPM7, and CNNM2 mRNAs [23]. SC and CC increased the mRNA levels of TRPM6 and CNNM2 without affecting that of TRPM7 (Figure 1). The SC-induced elevation of TRPM6 mRNA was significantly blocked by U0126, a MEK/ERK inhibitor (Figure 2A–C). In contrast, U0126 had no effect on the mRNA levels of CNNM2 and TRPM7. Western blot analysis showed that SC increased TRPM6 expression, which was blocked by U0126 (Figure 2D). Similar to those in real-time PCR, the protein levels of CNNM2 and TRPM7 were unchanged by U0126. The TRPM6 expression is up-regulated by a MEK/ERK/c-Fos pathway in NRK-52E cells [19]. SC increased the levels of p-ERK1/2 and p-c-Fos, which were inhibited by U0126 (Figure 2E). These results indicate that SC increases TRPM6 expression mediated by the activation of MEK/ERK pathway in NRK-52E cells.

### 3.2. Increase in TRPM6 Expression by Alkalization

**Figure 3:**
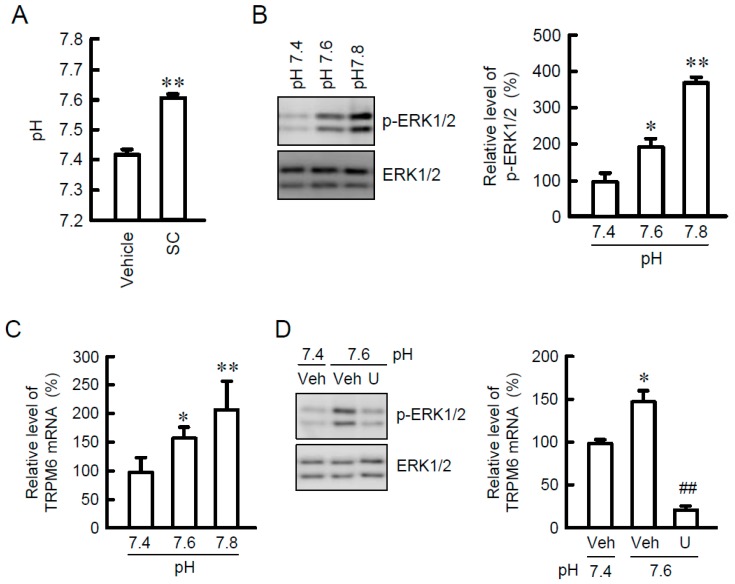
Increase in TRPM6 and p-ERK1/2 expression by SC. (**A**) The pH of culture medium was measured using a pH meter. (**B**) NRK-52E cells were incubated in the medium of indicated pH for 15 min. Cytoplasmic extracts were immunoblotted with anti-p-ERK1/2 or anti-ERK1/2 antibody. The expression levels were represented relative to the values at pH 7.4. (**C**) Cells were incubated in the medium of indicated pH for 24 h. Quantitative real-time PCR was performed using primer pairs of TRPM6 and β-actin. The contents of TRPM6 mRNA were represented as fold-increase over vehicle. (**D**) Cells were incubated in the medium of indicated pH for 15 min in the absence (veh) or presence of 10 μM U0126 (U). Cytoplasmic extracts were immunoblotted with anti-p-ERK1/2 or anti-ERK1/2 antibody. The expression levels were represented relative to the values at pH 7.4. *n* = 4. ** *p* < 0.01 and * *p* < 0.05 compared with pH 7.4. ^##^ *p* < 0.01 compared without U0126.

The pH of cell culture medium was alkalized by treatment with SC (Figure 3A). Similarly, CC induced alkalization of culture medium [23]. Alkalization of Hepes-buffered medium (pH 7.6 and 7.8) caused the activation of p-ERK1/2 (Figure 3B). Similarly, SC increased phosphorylation level of c-Fos [23]. The mRNA level of TRPM6 and phosphorylation level of ERK1/2 were increased by alkalization, which was blocked by U0126 (Figure 3C,D). The effect of U0126 on alkalization-induced changes coincide with that on SC-induced elevation of p-ERK1/2 and TRPM6 expression.

### 3.3. Effects of SC and Alkalization on Mg^2+^ Influx

**Figure 4:**
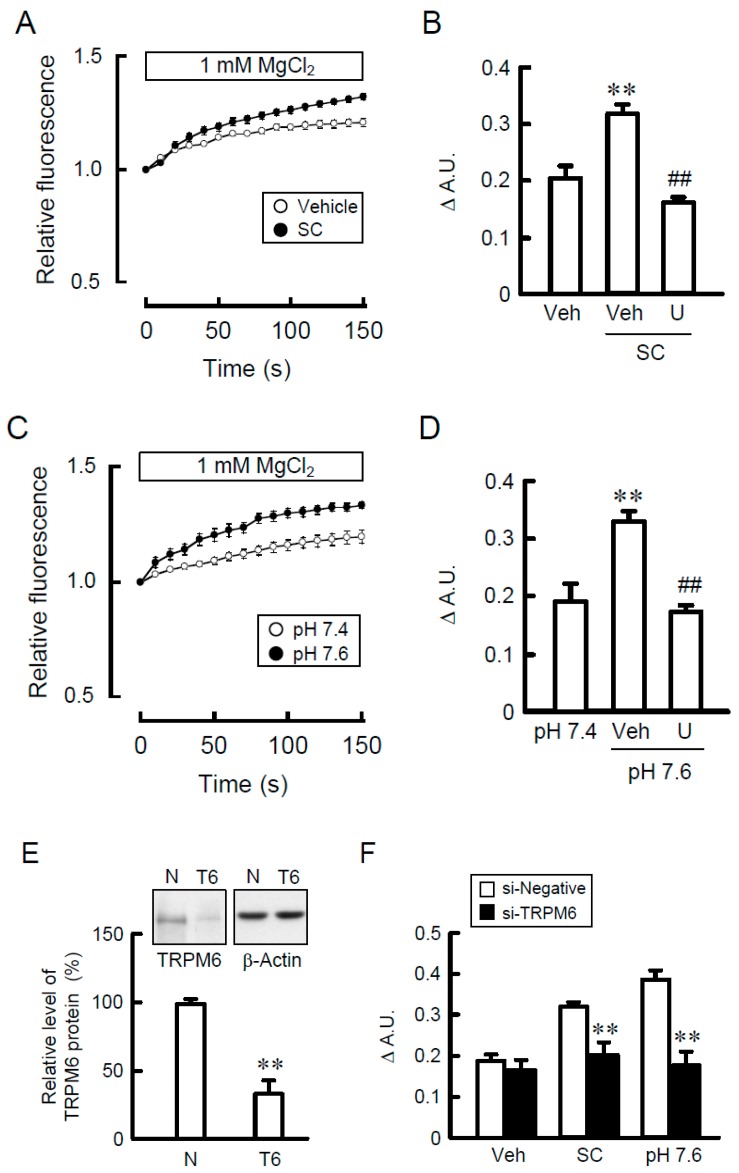
Enhancement of Mg^2+^ influx by SC. (**A**) NRK-52E cells were incubated with vehicle (open circles) or 10 μM SC (closed circles) for 24 h. MgCl_2_ was added to the nominally Mg^2+^-free HBSS immediately after measurement start. The fluorescence values of Mag-fura 2 are represented relative to the values measured at 0 s in the vehicle cells. (**B**) The increase in fluorescence values of Mag-fura 2 for 150 s was compared in each group. (**C**,**D**) Cells were incubated in the medium as indicated pH for 24 h in the absence (veh) or presence of 10 μM U0126. The increase in fluorescence values of Mag-fura 2 for 150 s was compared in each group. (**E**) Cells were transfected with negative (N) or TRPM6 siRNA (T6). Cytoplasmic extracts were immunoblotted with anti-TRPM6 or anti-β-actin antibody. The expression levels were represented relative to the values in negative siRNA. (**F**) Cells were transfected with negative or TRPM6 siRNA, and then incubated with vehicle (veh), 10 μM SC, or at pH 7.6 for 24 h. The increase in fluorescence values of Mag-fura 2 for 150 s was compared in each group. *n* = 3–4. ** *p* < 0.01 compared with vehicle, pH 7.4, or negative siRNA. ^##^ *p* < 0.01 compared without U0126.

To clarify whether TRPM6 is functionally expressed in NRK-52E cells, we examined the effects of SC and alkalization on Mg^2+^ influx. The addition of MgCl_2_ increased the fluorescence intensity of Mag-fura 2 ([Mg^2+^]_i_) in a time-dependent manner (Figure 4A). SC significantly enhanced the elevation of [Mg^2+^]_i_, which was blocked by U0126 (Figure 4B). Similarly, alkalization of culture medium enhanced the elevation of [Mg^2+^]_i_, which was blocked by U0126 (Figure 4C,D). Next, we investigated the effect of TRPM6 siRNA on [Mg^2+^]_i_,. The protein level of TRPM6 was reduced by more than 70% by the introduction of TRPM6 siRNA compared to negative siRNA (Figure 4E). The elevation of [Mg^2+^]_i_ caused by SC and alkalization was significantly inhibited by TRPM6 siRNA (Figure 4F). These results indicate that TRPM6 induced by SC may be functionally expressed in NRK-52E cells.

### 3.4. Effects of SC and Alkalization on TRPM6 Promoter Activity

**Figure 5:**
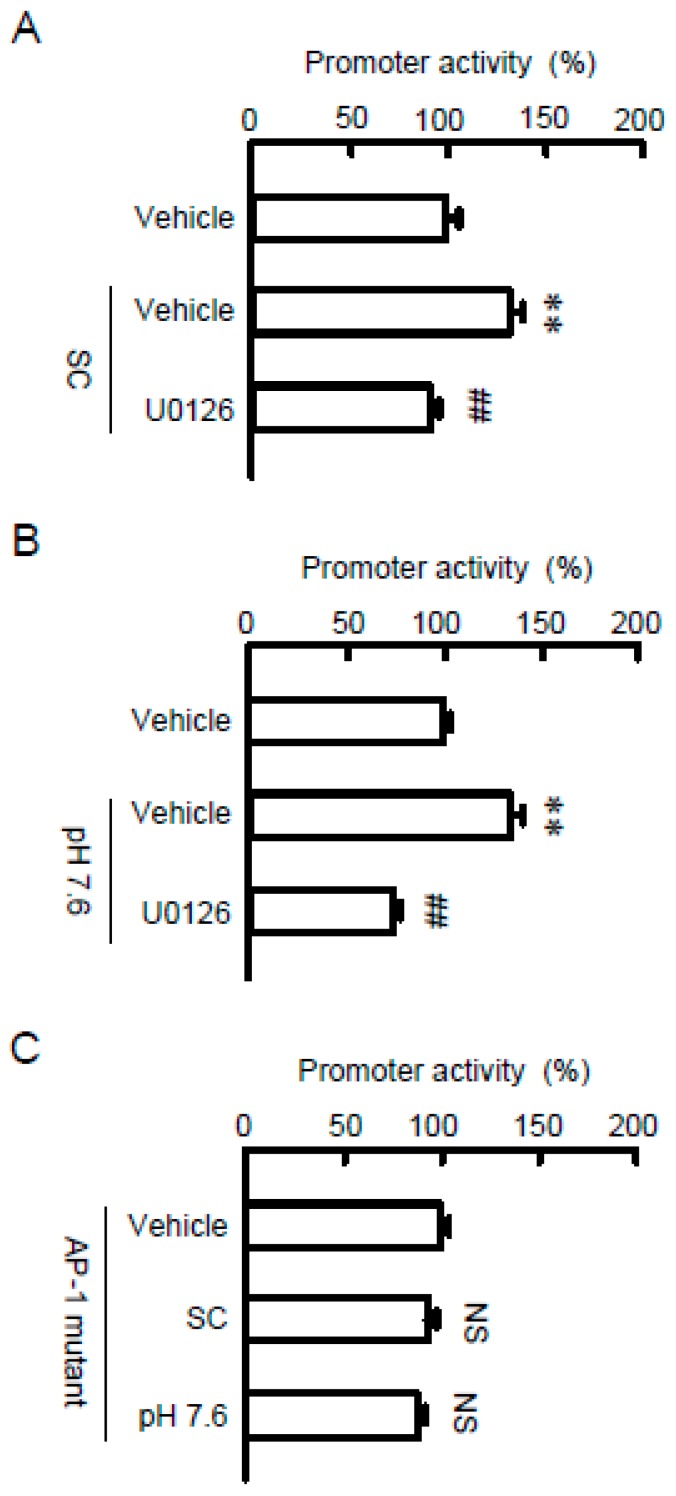
Involvement of AP-1 binding site on the SC-induced elevation of TRPM6 promoter activity. (**A**,**B**) A TRPM6 promoter luciferase vector was co-transfected with the pRL-TK vector into NRK-52E cells. At 24 h after transfection, the cells were incubated with vehicle, 10 μM SC (**A**), or at pH 7.6 (**B**) for 24 h. If needed, the cells were pre-incubated with 10 μM U0126 for 30 min. The relative promoter activity was represented as a percentage of vehicle. (**C**) Mutated construct of the AP-1 binding sites in TRPM6 promoter was co-transfected with the pRL-TK vector into NRK-52E cells. At 24 h after transfection, the cells were incubated with 10 μM SC or at pH 7.6 for 24 h. The relative promoter activity was represented as a percentage of vehicle. *n* = 4–5. ** *p* < 0.01 and NS *p* > 0.05 compared with vehicle. ^##^ *p* < 0.01 compared without U0126.

The promoter activity of TRPM6 is up-regulated by the MEK/ERK/c-Fos pathway in NRK-52E cells [19]. SC and alkalization increased the promoter activity, which was significantly blocked by U0126 (Figure 5A,B). The SC- and alkalization-induced elevation of promoter activity was inhibited by the mutation of AP-1 binding site in the promoter region of TRPM6 (Figure 5C). These results indicate that both SC and alkalization may increase the AP-1-dependent transcriptional activity of TRPM6.

### 3.5. Involvement of NADPH Oxidase in SC-Induced Elevation of TRPM6 Expression

**Figure 6:**
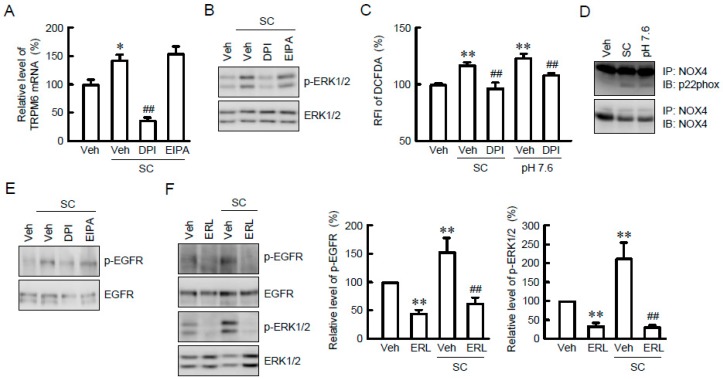
Activation of nicotinamide adenine dinucleotide phosphate (NADPH) oxidase and epidermal growth factor receptor (EGFR) by SC. (**A**) NRK-52E cells were incubated with vehicle (veh), 10 μM SC, 10 μM diphenyleneiodonium (DPI), or 10 μM 5-(*N*-ethyl-*N*-isopropyl) amiloride (EIPA) for 24 h. Quantitative real-time PCR was performed using primer pairs of TRPM6 and β-actin. (**B**) Cells were incubated with vehicle (vih), 10 μM SC, 10 μM DPI, or 10 μM EIPA for 15 min. Cytoplasmic extracts were immunoblotted with anti-p-ERK1/2 or anti-ERK1/2 antibody. (**C**) Cells were incubated with vehicle (veh), 10 μM SC, 10 μM DPI, or pH 7.6 for 15 min. ROS production was assessed by 2′,7′-dichlorofluorescin diacetate (DCFDA) fluorescence measurement and represented as a percentage of vehicle. (**D**) Cells were incubated with vehicle (veh), 10 μM SC or pH 7.6 for 15 min. Cytoplasmic extracts were immunoprecipitated with anti-NADPH oxidase (NOX) 4 antibody. After sodium dodecyl sulfate-polyacrylamide gel electrophoresis (SDS-PAGE), the membrane was blotted with anti-p22phox or anti-NOX4 antibody. (**E**,**F**) Cells were incubated with vehicle (veh), 10 μM SC, 10 μM DPI, 10 μM EIPA, or 10 μM erlotinib (ERL) for 15 min. Cytoplasmic extracts were immunoblotted with anti-p-EGFR, anti-EGFR, anti-p-ERK1/2, or anti-ERK1/2 antibody. The expression levels were represented relative to the values in vehicle. *n* = 3–4. ** *p* < 0.01 and * *p* < 0.05 compared with vehicle. ^##^ *p* < 0.01 compared without DPI or ERL.

It is unknown how SC activates the MEK/ERK pathway. The alkalization of culture medium has been reported to affect NADPH oxidase and Na^+^/H^+^-exchanger. Therefore, we examined the effect of specific inhibitor on these molecules. The SC-induced elevation of TRPM6 expression was significantly suppressed by DPI, an NADPH oxidase inhibitor, but not by EIPA, a Na^+^/H^+^-exchanger inhibitor (Figure 6A). Similarly, the activation of ERK1/2 caused by SC was blocked by DPI, but not by EIPA (Figure 6B). NADPH oxidase can produce ROS [24]. The fluorescence intensity of DCFDA, a maker of ROS production, was increased by SC and alkalization, which was blocked by DPI (Figure 6C). The activation of NADPH oxidase requires the association between NOX4 and p22phox [25]. In immunoprecipitation assay, the association of NOX4 with p22phox was augmented by SC and alkalization (Figure 6D). These results indicate that NADPH oxidase may be involved in the SC-induced elevation of TRPM6 expression. Next, we examined the involvement of EGFR in SC-induced ERK1/2 activation. EGFR was phosphorylated by SC, which was attenuated by DPI (Figure 6E). The SC-induced elevation of p-EGFR and p-ERK1/2 was significantly blocked by erlotinib, an EGFR tyrosine kinase inhibitor (Figure 6F). These results indicate that SC may activate the MEK/ERK pathway mediated by the phosphorylation of EGFR.

### 3.6. Association of c-Fos with the TRPM6 Promoter Region

**Figure 7:**
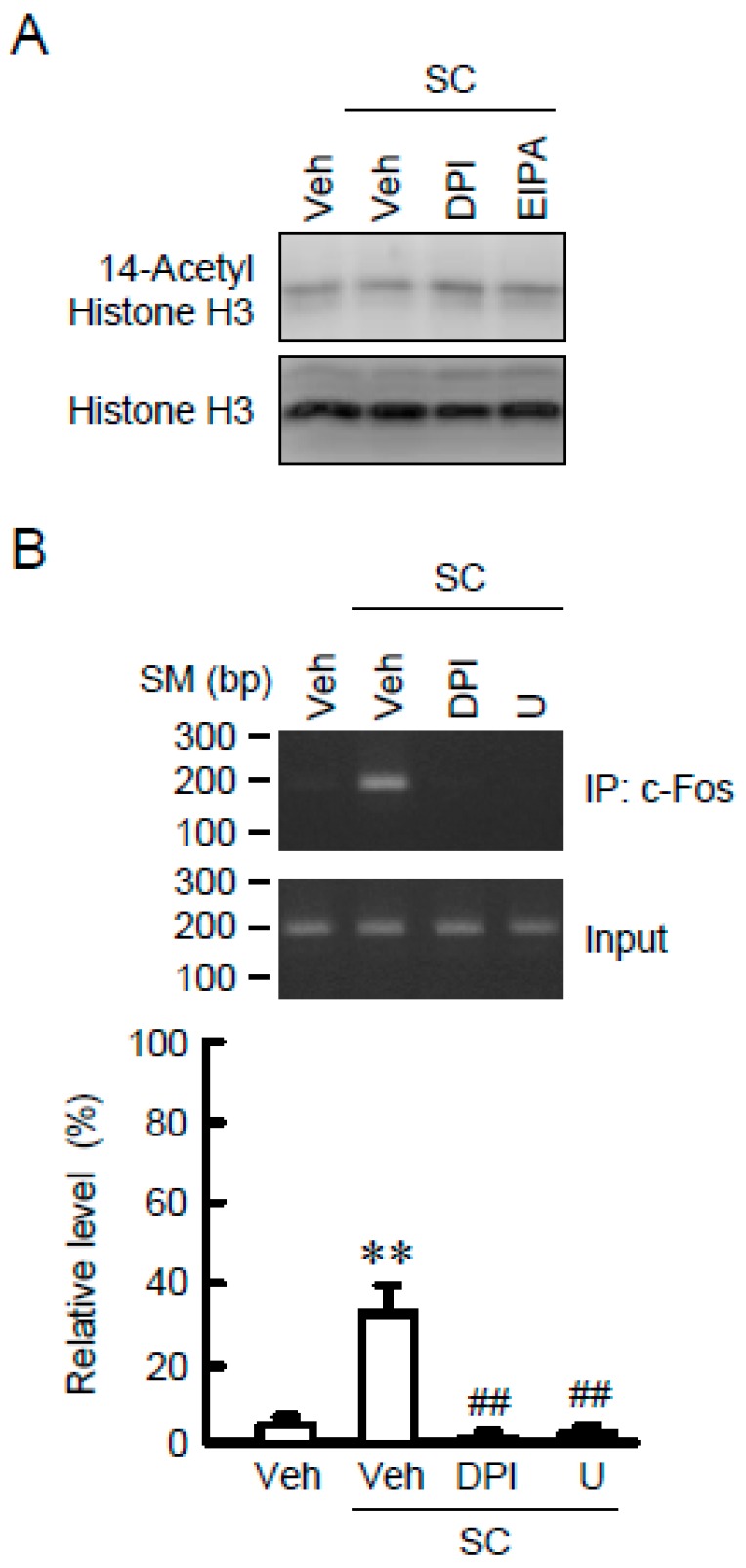
Association of c-Fos with AP-1 binding site in the TRPM6 promoter. (**A**) NRK-52E cells were incubated with vehicle (veh), 10 μM SC, 10 μM DPI, or 10 μM EIPA for 24 h. Nuclear extracts were immunoblotted with anti-acetyl-histone H3 or anti-histone H3 antibody. (**B**) Cells were incubated with vehicle (veh), 10 μM SC, 10 μM DPI, or 10 μM EIPA for 2 h. Genomic DNA was immunoprecipitated with an anti-c-Fos antibody. After immunoprecipitation, the 5′-flanking region of TNRP6 was amplified by semi-quantitative (upper gel photos) and quantitative PCR (lower graphs) using primer pairs amplifying AP-1 binding site. Input was amplified by the primer without immunoprecipitation. ChIP data are represented as % of input. *n* = 3. ** *p* < 0.01 compared with vehicle. ^##^ *p* < 0.01 compared with SC alone.

**Figure 8:**
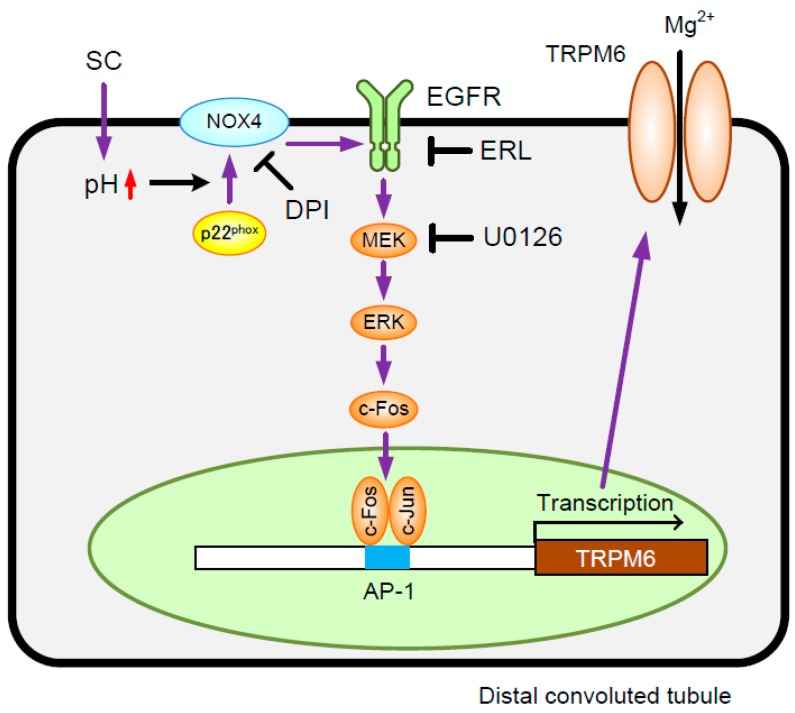
A putative scheme for the elevation of TRPM6 expression by SC. SC increases TRPM6 expression mediated by the activation of NOX4 and EGFR/mitogen-activated protein kinase kinase (MEK) /extracellular signal-regulated kinase (ERK)/c-Fos/AP-1 pathway, resulting in the elevation of Mg^2+^ influx. The SC-induced elevation of TRPM6 expression and Mg^2+^ influx is blocked by DPI, ERL, or U0126.

Recently, the transcriptional regulation of various genes is reported by epigenetic alterations such as histone modification [26]. The acetylation level of histone H3 was unchanged by SC, DPI, and EIPA (Figure 7A), indicating that epigenetic alteration may be not involved in the SC-induced elevation of TRPM6 expression. Next, the association of c-Fos, a downstream transcriptional effector of MEK/ERK signaling, with the AP-1 binding site of TRPM6 promoter was examined. In the ChIP assay, a primer pair -421S/-616A, which amplifies the region containing AP-1 binding site, showed no PCR signals in vehicle-treated cells (Figure 7B). In contrast, PCR signals were detected in SC-treated cells, which was blocked by DPI and U0126. In the input images, similar PCR signals were detected in all samples. These results indicate that SC up-regulates the association of c-Fos with the TRPM6 promoter region. A tentative model is described in Figure 8.

## 4. Discussion

It is little known how the gene expression of *TRPM6* and *CNNM2* is regulated in renal tubular epithelial cells. So far, we reported that epidermal growth factor increases the expression levels of TRPM6 mediated by the activation of MEK/ERK/c-Fos pathway using renal epithelial NRK-52E cells [19,20]. Immunosuppressants including cyclosporine A and tyrosine kinase inhibitor erlotinib, an anticancer drug used in the treatment of non-small lung cancer, induce the reduction of TRPM6 expression mediated by the inhibition of MEK/ERK/c-Fos pathway [21,22]. Therefore, the MEK/ERK/c-Fos pathway may play a pivotal role in Mg^2+^ homeostasis under physiological conditions. In the present study, we found that SC increases the expression level of TRPM6 mediated by the activation of MEK/ERK/c-Fos pathway. In contrast, SC could elevate the expression level of CNNM2, but the phenomenon was not inhibited by U0126. CNNM2 may be up-regulated by SC mediated through a different mechanism from that of TRPM6 in NRK-52E cells. A low Mg^2+^ diet increases the gene expression of CNNM2 in the mice [14]. Furthermore, a low Mg^2+^-containing medium increases CNNM2 levels in mouse DCT cells. Therefore, CNNM2 is also suggested to play an important role in the improvement of Mg^2+^ deficiency. Further study is necessary to clarify the regulatory mechanism of CNNM2 expression.

SC is commonly utilized as a chelating agent, emulsifier, and pH adjuster in many food applications. Sodium and citrate feature a high degree of safety because they are abundantly contained in various foods. In clinical therapy, SC is widely used as prevention and treatment of calcium oxalate stones. Serum citrate is filtrated at the glomeruli and then reabsorbed from renal tubules about 65–90% [27]. Only 10–35% of the filtrated citrate is excreted into urine. The majority of filtrated citrate may be reabsorbed by a sodium-dicarboxylate co-transporter (NaDC-1), which is localized in the apical membrane, in the proximal tubules [28]. It is unclarified whether NaDC-1 are expressed in the DCT. Therefore, we cannot deny the possibility that intracellular citrate is involved in the up-regulation of TRPM6 expression although extracellular alkalization increased TRPM6 expression similar to SC. Mg^2+^-citrate transporter (CitM) has been identified in *Bacillus subtilis* and *E. coli* DH5 cells. A mammalian subtype of CitM has not been cloned and we do not know whether CitM is expressed in mammalian renal tubules. However, the involvement of CitM in the SC-induced elevation of Mg^2+^ influx in NRK-52E cells may be excluded because the function of CitM is inhibited in buffers that are more alkaline than pH 7.5 [29].

There is a case report showing that the renal wasting of Mg^2+^ and Ca^2+^ in a patient with nephrolithiasis is improved by being treated with Mg^2+^ and Ca^2+^ supplementation plus citrate salts [30]. Mild hypomagnesemia developed in subjects treated with short-term thiazide is corrected by potassium-magnesium citrate supplementation [31]. These reports support our data that SC increases Mg^2+^ influx in NRK-52E cells. In animal models, metabolic acidosis, which is induced by oral NH_4_Cl loading, induces reduction of TRPM6 mRNA levels and enhances urinary Mg^2+^ excretion [32]. In contrast, metabolic alkalosis, which is induced by NaHCO_3_ treatment, induces opposite effects. Micropuncture studies showed that metabolic alkalosis increases Mg^2+^ transport in the DCT [33]. Urinary acid-base status should be closely related to the regulation of TRPM6 expression, but the mechanism has not been fully clarified. In the present study, we found that alkalosis, which is induced by SC or Hepes buffer, increases the mRNA level, protein level, and reporter activity of TRPM6 in NRK-52E cells. SC increased the phosphorylation of EGFR, ERK, and c-Fos, which were completely blocked by U0126. In addition, the SC-induced elevation of TRPM6 expression was blocked by U0126 and erlotinib. We suggest that SC increases TRPM6 expression mediated via the activation of EGFR/MEK/ERK/c-Fos pathway. This is the first report showing that SC, one of the food components contained in fruits, milks, and beans, increases TRPM6 expression in renal tubular NRK-52E cells. However, the data are limited in cultured cells. We need further study as to whether SC ameliorates Mg^2+^ deficiency using animal model.

Some acid-sensing mechanisms have been identified including acid/alkali-sensing receptor, kinases, pH-sensitive ion channels, and the bicarbonate-stimulated adenylyl cyclase [34]. Extracellular alkalosis activates the EGFR/ERK pathway mediated via a NOX-dependent ROS production [35,36]. NOX is divided into five isoforms; NOX1-NOX5. Among them, NOX4 is most abundantly expressed in the kidney [37]. The protein expression of NOX4 in the DCT is revealed by immunostaining [38]. Both SC and alkalization increased the association of NOX4 with p22phox and the production of ROS (Figure 6C,D). Our data indicate that SC increases p-EGFR and p-ERK1/2 levels, which were blocked by DPI, suggesting that NOX activates the MEK/ERK/c-Fos pathway via the activation of EGFR in NRK-52E cells. This assumption is supported by the fact that SC-induced elevation of p-EGFR and p-ERK, which is blocked by EGFR tyrosine kinase inhibitor erlotinib.

## 5. Conclusions

In the present study, we found that SC increases TRPM6 expression and Mg^2+^ influx mediated by the activation of NADPH oxidase and EGFR/ERK/AP-1 pathway in NRK-52E cells. While the regulatory mechanism of CNNM2 expression was unclarified, SC increased the mRNA and protein levels of CNNM2. These results suggest that SC increases TRPM6-mediated Mg^2+^ influx in the apical membrane and CNNM2-mediated Mg^2+^ efflux in the basolateral membrane, resulting in the elevation of transcellular Mg^2+^ reabsorption in the DCT. SC may be a novel magnesiotropic food factor for preventing chronic Mg^2+^ deficiency.

## Figures and Tables

**Table 1:** Primer lists.

Primer Name	Primer Sequence
Rat TRPM6	Sense: 5′-CTTCTTGGGATACCAAATCAG-3′
	Antisense: 5′-GAAACTTTTCCTAGTGTAGCTG-3′
Rat TRPM7	Sense: 5′-AACCAACACTCTGGAAGAGATCA-3′
	Antisense: 5′-TCAGTCAAGTTTTCTCCCACAC-3′
Rat CNNM2	Sense: 5′-AACACCATCTTCCTCACCAAGT-3′
	Antisense: 5′-TCAGCTCTTCCTTAACGAGGTC-3′
Rat β-actin	Sense: 5′-CCAACCGTGAAAAGATGACC-3′
	Antisense: 5′-CCAGAGGCATACAGGGACAG-3′
Human TRPM6 (hTRPM6)	Sense: 5′-AAAGTTCAATTGGAGTTGACAAGA-3′
	Antisense: 5′-AAATTATTCCTTTCAATGGCTGA-3′
-421S/-616A	Sense: 5′-CTGTGTGCTTTGTGCCACCTC-3′
	Antisense: 5′-GAAATGGGGTCTCACTATGTTG-3′

## Abbreviations

CC	calcium citrate
ChIP	chromatin immunoprecipitation
CitM	Mg^2+^-citrate transporter
CNNM2	cyclin M2
Ct	threshold cycle
DCFDA	2′,7′-dichlorofluorescin diacetate
DCT	distal convoluted tubule
DPI	diphenyleneiodonium
EGFR	epidermal growth factor receptor
EIPA	5-(*N*-ethyl-*N*-isopropyl) amiloride
ERK	extracellular signal-regulated kinase
HBSS	Hank’s balanced salt solution
Mag-fura-2 AM	2-[5-[2-[(acetyloxy)methoxy]-2-oxoethoxy]-6-[bis[2-[(acetyloxy)methoxy]-2-oxoethyl]amino]-2-benzofuranyl]-(acetyloxy)methyl ester
Mg^2+^	magnesium ion
[Mg^2+^]_i_	intracellular free Mg^2+^ concentration
MEK	mitogen-activated protein kinase kinase
NaDC	sodium-dicarboxylate co-transporter
NADPH	nicotinamide-adenine dinucleotide phosphate
NOX	NADPH oxidase
PCR	polymerase chain reaction
p-ERK1/2	phosphorylated ERK1/2
PVDF	polyvinylidene fluoride
SC	sodium citrate
SDS-PAGE	sodium dodecyl sulfate-polyacrylamide gel electrophoresis
TAL	thick ascending limb of Henle’s loop
TRPM6	transient receptor potential melastatin 6
